# Disrupted immune senescence in early pregnancy is associated with recurrent pregnancy loss

**DOI:** 10.3389/fimmu.2026.1705823

**Published:** 2026-02-04

**Authors:** Dimitar Parvanov, Rumiana Ganeva, Margarita Ruseva, Maria Handzhiyska, Lachezar Jelezarsky, Jinahn Safir, Dimitar Metodiev, Georgi Stamenov, Savina Hadjidekova

**Affiliations:** 1 Research, Nadezhda Women’s Health Hospital, Sofia, Bulgaria; 2 Clinical Pathology, Nadezhda Women’s Health Hospital, Sofia, Bulgaria; 3 Obstetrics and Gynecology, Nadezhda Women’s Health Hospital, Sofia, Bulgaria; 4 Medical Genetics, Medical University of Sofia, Sofia, Bulgaria; 5 Medical Genetics, Nadezhda Women’s Health Hospital, Sofia, Bulgaria

**Keywords:** biomarkers, cell cycle regulators, early pregnancy, flow cytometry, immune senescence, maternal-fetal tolerance, peripheral blood leukocytes, recurrent pregnancy loss

## Abstract

**Background:**

Immune system adaptation plays a crucial role in pregnancy success. Cellular senescence, linked to immune dysfunction and chronic inflammation, may contribute to adverse pregnancy outcomes but remains poorly characterized during early gestation, at the systemic level.

**Objective:**

To characterize senescence marker expression in peripheral leukocyte subsets in early pregnancy and to examine its association with miscarriage history and functionally distinct T helper cell populations.

**Methods:**

This cross-sectional study included 52 pregnant women: 18 with recurrent pregnancy loss (≥2 miscarriages), 18 with one prior loss, and 16 controls with no loss history. Peripheral blood leukocytes were analyzed using an automated hematology analyzer and by flow cytometry for senescence markers (p16, p21, p53) and major T-cell subsets. Group differences were tested by ANOVA or Kruskal–Wallis test, as appropriate and exploratory Spearman correlation analyses examined associations between miscarriage number, senescent subsets, and T-cell populations.

**Results:**

Senescent cells were detected as percentages of granulocytes, monocytes, and lymphocytes, with granulocytes exhibiting the highest senescence expression. A consistent p21>p16>p53 expression pattern across immune subsets was observed in controls, suggesting a physiologic senescence hierarchy. This organization was progressively disrupted in miscarriage groups. A stepwise increase with miscarriage number was observed across subsets. Significant differences were observed in the median percentage of p16^+^ monocytes between controls, one prior loss and RPL patients (0.20%, 0.41%, and 0.57%, respectively; p = 0.03). Corresponding increases were also detected for p16^+^ lymphocytes (0.06%, 0.17%, 0.35%; p < 0.01), p21^+^ granulocytes (0.78%, 5.75%, 11.7%; p = 0.04), and p53^+^ granulocytes (4.01%, 7.05%, 8.35%; p = 0.05). Spearman analyses supported these trends and further revealed positive associations between p16^+^ monocytes and Th17 cells and between p53^+^ granulocytes and Th1 cells (p < 0.05).

**Conclusion:**

Pregnancy loss history is associated with both quantitative and qualitative alterations in immune senescence profiles, including organizational remodeling of peripheral immune senescence. Analysis of proinflammatory and regulatory T-cell subsets revealed additional associations, suggesting immune senescence may interact with adaptive immune polarization in early pregnancy. These findings highlight disrupted immune senescence as a potential component of immune maladaptation and identify senescent immune cells as candidate biomarkers for recurrent miscarriage.

## Introduction

Pregnancy success depends on maternal immune adaptation that permits tolerance to the semi-allogeneic fetus while maintaining protection against infection. Disruption of this delicate immune balance is a recognized contributor to early pregnancy loss (1–3). While considerable attention has been devoted to pro-inflammatory cytokines, NK cells, and Treg dysfunction in recurrent miscarriage (RPL) (4), the potential role of immune senescence remains largely unexplored.

Cellular senescence, defined as a state of stable cell cycle arrest accompanied by distinct secretory and metabolic changes, has emerged as a central component of immune aging and chronic inflammation (5, 6). Senescent immune cells often display a senescence-associated secretory phenotype (SASP), secreting IL-6, TNF-α, and other mediators that may disrupt tissue homeostasis (7, 8). This phenotype, while classically linked to aging, may also emerge prematurely in pathological states including autoimmunity and pregnancy complications (9, 10).

At the molecular level, senescence in immune cells is coordinated by two intersecting pathways, both measurable in peripheral blood — p53-p21 axis, which initiates growth arrest, and the p16–RB pathway, which maintains cell cycle inhibition (11). These pathways are reflected in classical senescence markers - p16^INK4a, p21, and p53 linked to DNA damage, oxidative stress, and cell cycle inhibition (12). Their expression in immune cells during early gestation may signal maladaptive immune aging, particularly in women with a history of miscarriage. However, no single biomarker uniformly captures senescence across immune lineages and blood-based work supports cell subset based, multimarker assessments (11, 13).

Despite increasing interest in immunosenescence, few studies have investigated its presence and organization during pregnancy, and none have quantitatively assessed its relationship to prior pregnancy loss. Most existing research has focused on tissue-level senescence at the maternal–fetal interface. For example, decidual senescence has been shown to influence implantation dynamics (14, 15), and excessive p53/p21-driven senescence in decidual stromal cells has been associated with recurrent miscarriage (16). Our previous work has also demonstrated that senescent cells in the luminal epithelium display specific patterns associated with successful implantation, further highlighting the importance of localized senescence at the uterine level (17). More recently, we reported that endometrial immune cell populations display specific associations with senescence cells, further underscoring the importance of coordinated epithelial–immune interactions in reproductive success (18). However, while these findings underscore the importance of localized uterine senescence, they are largely limited to tissue-level observations and offer little insight into circulating immune cells. In this context, peripheral blood immune profiling provides complementary information by capturing systemic immune aging, inflammatory bias, and long-term immune programming that may influence immune cell recruitment and function at the maternal–fetal interface, thereby contextualizing local uterine immune responses. Thus, comprehensive quantitative analysis of peripheral immune senescence in women with a history of miscarriage remains unexplored.

In this study, we assessed the expression of senescence markers in peripheral blood leukocyte subsets (granulocytes, monocytes, and lymphocytes) during the first trimester of pregnancy. In addition to characterizing senescence profiles in women with and without prior pregnancy loss, we investigated their relationship with key T helper cell subsets (Th1, Th2, Th17, TFH, Treg), given their established roles in reproductive immunology and inflammation. Senescent immune cells may influence adaptive immune polarization through cytokine secretion or checkpoint signaling, and conversely, proinflammatory T cells may promote senescence via sustained immune activation. Therefore, mapping these potential associations may help uncover coordinated maladaptive changes within the maternal immune system. Accordingly, T-cell subset analysis was included as a complementary approach to contextualize senescence-associated findings within broader immune network alterations, rather than as a parallel primary endpoint.

Our goal was to determine whether immune senescence profiles are altered in association with previous pregnancy loss, whether they follow a pattern reflecting cumulative reproductive stress, and how they relate to specific T-cell phenotypes in early pregnancy.

## Materials and methods

### Study design and participants

This observational cross-sectional study was designed to evaluate immune cell senescence in early pregnancy in relation to pregnancy loss history. All participants were enrolled during the first trimester of pregnancy (gestational age 6–12 weeks). The study was conducted in accordance with the Declaration of Helsinki and approved by the institutional ethics committee. Informed consent was obtained from all participants prior to sample collection.

Pregnancy loss was defined as a clinically recognized intrauterine pregnancy, typically confirmed by ultrasound and, where applicable, documented fetal cardiac activity, followed by subsequent pregnancy failure. Very early biochemical losses and pregnancies lost before detection of fetal heart activity (<5 gestational weeks) were not included. Detailed etiological classification of previous pregnancy losses (e.g., chromosomal abnormalities, structural fetal anomalies, or specific maternal causes) was not uniformly available or consistently documented for all participants and was therefore not used for stratification. As such, prior pregnancy losses were analyzed as a clinical entity based on obstetric history rather than on confirmed underlying etiology.

Participants were stratified into three groups based on their reproductive history and confirmed by medical records: (1) Control group (n = 16): pregnant women with no history of miscarriage. (2) One-loss group (n = 18): pregnant women with a history of one prior spontaneous miscarriage. (3) Recurrent pregnancy loss (RPL) group (n = 18): pregnant women with two or more previous spontaneous miscarriages, meeting the ASRM criteria for RPL (19).

### Inclusion criteria

Pregnant women aged 28 to 50 years were enrolled from an IVF clinic between August 2023 and August 2025. Eligibility criteria included a positive serum or urine hCG test and confirmation of an intrauterine pregnancy with detectable fetal cardiac activity via transvaginal ultrasound, corresponding to 6–12 weeks of gestation.

Women were excluded if they had any of the following conditions: chronic inflammatory or autoimmune disease, known metabolic or endocrine disorders (e.g., diabetes, thyroid dysfunction), malignant disease, current immunosuppressive therapy, or recent invasive procedures.

Active infection at the time of inclusion was defined based on clinical assessment, including medical history, routine clinical evaluation, and the absence of symptoms or signs suggestive of acute infectious disease. Women with suspected or documented acute infection were excluded.

### Blood collection and processing

Peripheral venous blood samples (3 mL) were collected from each participant into EDTA-coated vacutainer tubes. Within two hours of collection, complete blood counts including leukocyte differentials (granulocytes, lymphocytes, monocytes) were performed using an automated hematology analyzer (Sysmex XN-1000, Sysmex Corporation, Kobe, Japan).

For senescence marker analysis, leukocytes were isolated by two-layer density gradient centrifugation using Pancoll human for granulocytes (P04-60150, density 1.119 g/mL, PAN-Biotech, Germany), which allows simultaneous separation of peripheral blood mononuclear cells (PBMCs) and granulocytes. Following centrifugation at 600 × g for 5 minutes at room temperature, the PBMC and granulocyte layers were carefully harvested, washed twice in phosphate-buffered saline (PBS) with 5% fetal bovine serum (FBS), and counted using an automated cell counter (Countess II FL, Thermo Fisher Scientific). Approximately 1 × 10^6^ cells were used for each antibody staining panel. All samples were processed under sterile conditions in a Class II biosafety cabinet and kept at 4°C until staining.

### Flow cytometry

**Figure 1:**
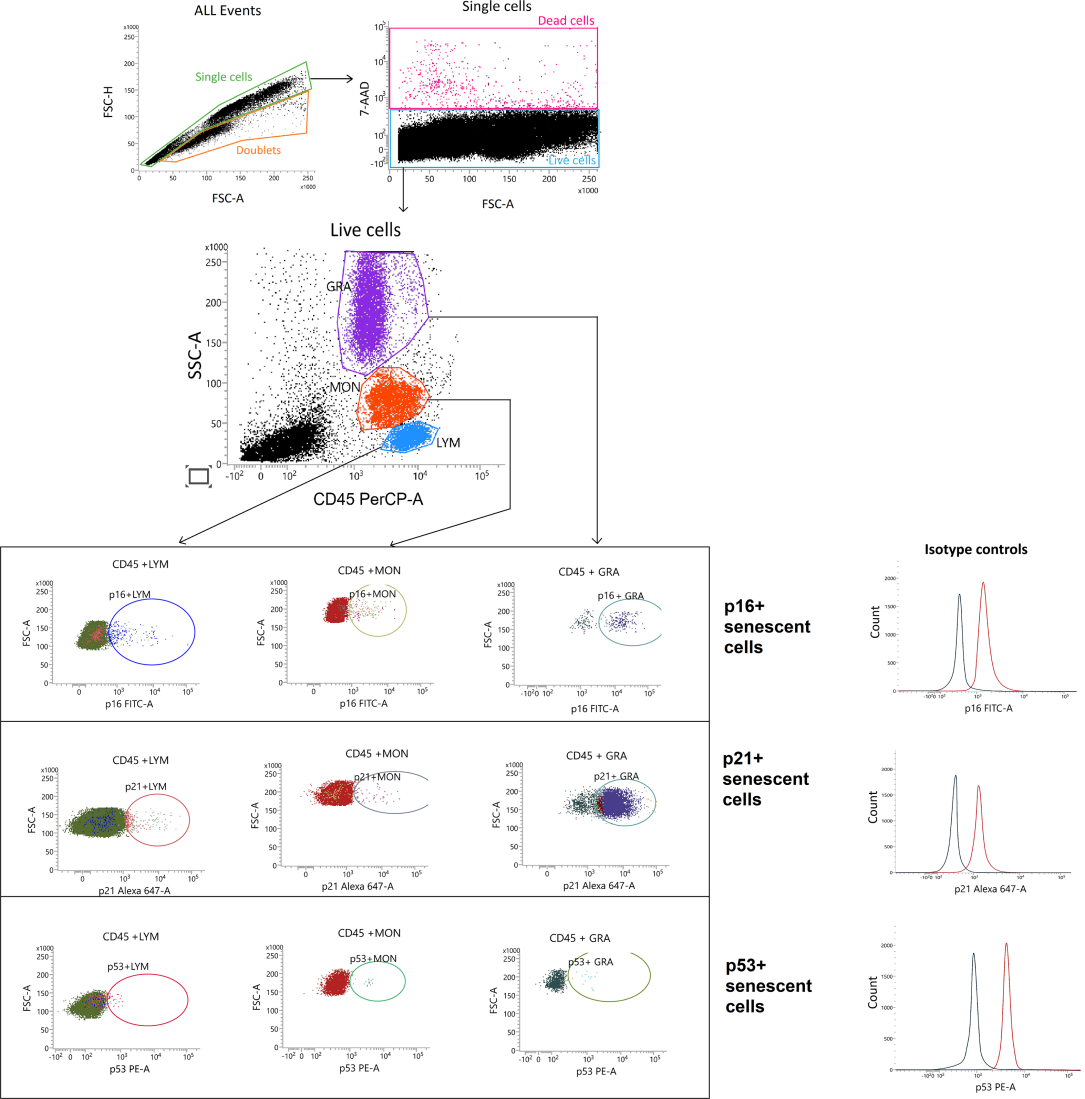
Flow cytometry gating strategy for identification of senescent leukocyte subpopulations. Representative gating strategy illustrating exclusion of doublets (FSC-A vs FSC-H) and dead cells (7-AAD), followed by identification of major leukocyte subsets. Live single leukocytes were identified based on CD45 expression and side scatter (SSC) characteristics, allowing discrimination of lymphocytes (LYM; CD45^bright/SSC^low), monocytes (MON; CD45^bright/SSC^intermediate), and granulocytes (GRA; CD45^dim/SSC^high). Senescent cells within each subset were detected by intracellular staining for p16 (FITC), p21 (Alexa Fluor 647), and p53 (PE) after fixation and permeabilization. Representative dot plots and histogram overlays show marker expression and isotype control staining.

Leukocyte senescence marker expression was assessed by flow cytometry using a BD FACS Lyric™ flow cytometer (BD Biosciences). Total leukocytes were identified using CD45-PerCP (Cat# 21810455, ImmunoTools). Leukocyte subsets were identified using a combined CD45 versus side scatter (SSC) gating strategy, allowing robust discrimination of lymphocytes (CD45bright/SSClow), monocytes (CD45bright/SSCintermediate), and granulocytes (CD45dim/SSChigh), in accordance with established flow cytometry conventions (**Figure 1**). After surface marker staining, cells were fixed and permeabilized using the BD Cytofix/Cytoperm™ kit (BD Biosciences) following the manufacturer’s protocol to allow for intracellular detection of senescence markers. The following monoclonal antibodies were used for intracellular staining: p16-FITC (Cat# 556560, BD Biosciences), p21-Alexa Fluor 647 (Cat# sc-6246, Santa Cruz Biotechnology), p53-PE (Cat# 645806, BioLegend).

Samples were incubated with antibodies at 4°C for 15 minutes in the dark. Appropriate isotype controls were included for all intracellular markers to assess background staining. A minimum of 100,000 events were acquired per sample. Peripheral blood samples were processed within two hours of collection. Viability staining was performed using 7-AAD (Cat# 130-111-565, Miltenyi Biotec) prior to fixation, allowing exclusion of dead cells from downstream analysis. Doublets were excluded using FSC-A versus FSC-H gating. Compensation was performed using single-stained controls.

**Figure 2:**
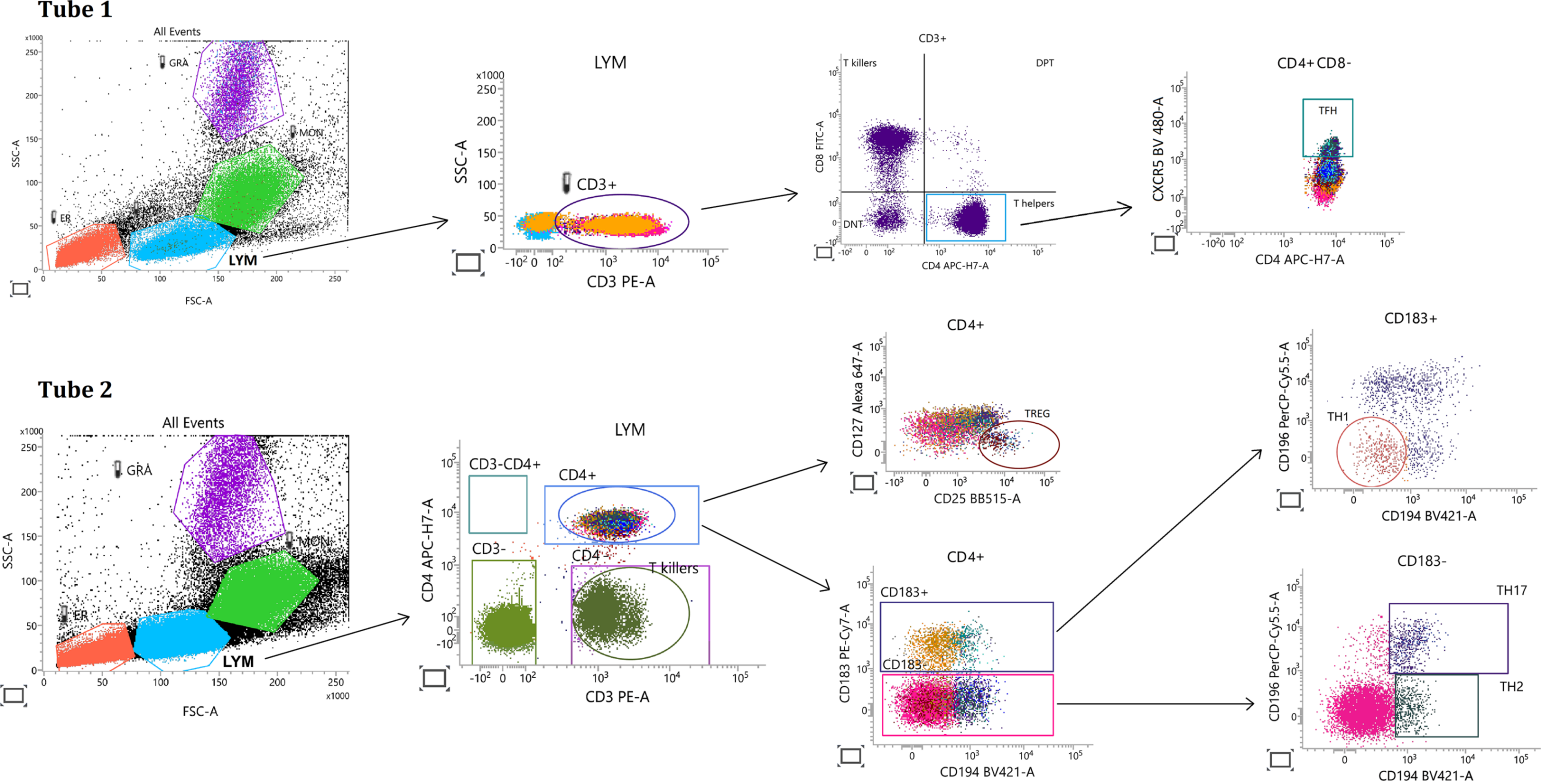
Flow cytometry gating strategy for identification of T-cell subsets in peripheral blood. Representative gating strategy illustrating initial selection of lymphocytes (LYM), followed by identification of CD3^+^ T cells and separation into CD4^+^ helper T cells and CD8^+^ cytotoxic T cells. Within the CD4^+^ population, functionally distinct T-helper subsets were defined based on chemokine receptor expression: Th1 (CD183^+^CD196^-^CD194^-^), Th2 (CD183^-^CD196^-^CD194^+^), and Th17 (CD183^-^CD196^+^CD194^+^). T follicular helper (TFH) cells were identified as CD4^+^CXCR5^+^ (CD185^+^), and regulatory T cells (Tregs) as CD4^+^CD25^+^CD127^-^. T-cell subsets were analyzed using separate antibody panels (Tube 1 and Tube 2), with the CD3^+^CD4^+^ lymphocyte population used as a gating reference across tubes, to minimize fluorochrome spectral overlap and ensure optimal subset resolution.

In addition to senescence profiling, phenotyping of major lymphocyte subsets was performed on the same freshly isolated peripheral blood samples (**Figure 2**). T helper cells (CD3^+^CD4^+^), and cytotoxic T cells (CD3^+^CD4^-^CD8^+^) were identified. Further T helper subsets were defined based on chemokine receptor expression: Th1 cells (CD4^+^CD183^+^CD196^-^CD194^-^), Th2 cells (CD4^+^CD183^-^CD194^+^CD196^-^), and Th17 cells (CD4^+^CD183^-^CD194^+^CD196^+^), T follicular helper cells (TFH) (CD3^+^CD4^+^ CD185^+^) and T regulatory cells (CD3^+^CD4^+^CD25^+^CD127^-^). The following monoclonal antibodies were used for this immunophenotyping: CD4–APC-H7 (Cat# 560158, BD Biosciences), CD8-FITC (Cat# 340692, BD Biosciences), CD25–BB515 (Cat# 564467, BD Biosciences), CD196 (CCR6)–BB700 (Cat# 566477, BD Biosciences), CD183 (CXCR3)–PE-Cy7 (Cat# 560831, BD Biosciences), CXCR5 BV480 (Cat# 566142, BD Biosciences), CD127–Alexa Fluor 647 (Cat# 558598, BD Biosciences), CD194 (CCR4)–BV421 (Cat# 562579, BD Biosciences). TFH and other helper subsets were acquired in separate panels, using the CD3^+^CD4^+^ lymphocyte population as a common gating reference.

### Statistical analysis

All statistical analyses were performed using SPSS version 21.0 (IBM Corp., Chicago, IL, USA). Graphs and visualizations were prepared using SPSS and R version 4.4.2 with the base graphics and ggplot2 packages. Descriptive statistics are presented as median and interquartile range (IQR) for non-normally distributed variables. Normality was assessed using the Shapiro–Wilk test. Comparisons between the three study groups (control, one prior miscarriage, and RPL) were performed using one-way analysis of variance (ANOVA) or Kruskal–Wallis test, depending on data distribution. For significant overall group differences, *post hoc* pairwise comparisons were conducted using the Least Significant Difference (LSD) test. A p-value < 0.05 was considered statistically significant.

To evaluate the adequacy of the sample size, a *post hoc* power analysis was conducted using G*Power software (version 3.1.9.7) and confirmed in R using the pwr package. The observed effect sizes and sample sizes yielded statistical power values (1–β) > 0.80, indicating sufficient sensitivity to detect differences in senescence marker expression.

Two separate exploratory correlation analyses were performed using Spearman’s rank correlation coefficient. The first assessed the relationship between the number of previous miscarriages and the proportions of senescent immune cells (p16^+^, p21^+^, and p53^+^) within granulocytes, monocytes, and lymphocytes. The second correlation matrix evaluated the associations between senescent immune subsets and the abundance of specific T cell subpopulations. These analyses were performed in R version 4.4.2 using the corrplot package for visualization. A two-tailed p-value < 0.05 was considered statistically significant.

Given the exploratory and hypothesis-driven nature of these analyses, correlation testing was restricted to a limited, predefined set of biologically relevant associations. Therefore, no formal correction for multiple testing was applied. This approach was chosen to preserve interpretability while minimizing the risk of type II error in this focused analysis.

## Results

Baseline characteristics, including age, BMI, and major leukocyte subset distribution, were first evaluated across the three groups (**Table 1**). No statistically significant differences were found between the studied groups, except for total granulocytes, which were significantly lower in both pregnancy loss groups compared to controls (**Table 1**).

**Table 1:** Baseline characteristics of the studied patient groups, stratified by number of previous miscarriages (median, IQR).

Variable	Total	Studied patient groups			P value
		Group 1 No Loss (n = 16)	Group 2 1 Loss (n = 18)	Group 3 RPL (≥2 Losses), n = 18)	
Age, years	39 (11)	40 (11)	38 (12)	38 (13)	0.85
BMI, kg/m²	23.6 (1.65)	23.5 (1.08)	23.7 (2.63)	23.7 (1.53)	0.93
Granulocyte count (×10^9^/L)	6.93 (3.95)	9.97 (10.10)	6.48 (4.72)	6.09 (1.69)	0.002*
Monocyte count (×10^9^/L)	0.58 (0.25)	0.62 (0.21)	0.54 (0.23)	0.48 (0.27)	0.07
Lymphocyte count (×10^9^/L)	1.70 (0.81)	1.84 (0.68)	1.57 (1.19)	1.70 (0.81)	0.51

The asterisk (*) indicates a statistically significant difference between study groups, defined as p < 0.05.

Expression of senescence markers was detected across all leukocyte subtypes, with variable frequency and intensity (**Figure 1**). Granulocytes exhibited the highest proportion of p16+ cells, followed by monocytes and lymphocytes (p < 0.05) (**Table 2**). This hierarchical pattern was not observed for p21 or p53 expression. In the control group, senescence marker distribution followed a consistent relationship: p21+ > p16+ > p53+, across all immune subsets (p < 0.05), suggesting a physiological senescence hierarchy in early pregnancy. In contrast, this organization was progressively altered in women with prior pregnancy loss.

**Table 2:** Senescent immune cell subsets and T-helper populations in peripheral blood of pregnant women with no pregnancy loss, one prior loss, or recurrent pregnancy loss (RPL).

Variable	Total	Studied patient groups			p value
		Group 1 No Loss (n = 16)	Group 2 1 Loss (n = 18)	Group 3 RPL (≥2 Losses), n = 18)	
p16+ Lymphocytes, %	0.15 (0.28)	0.06 (0.06)	0.17 (0.18)	0.35 (0.53)	< 0.001*
p16+ Monocytes, %	0.36 (0.73)	0.2 (0.27)	0.41 (0.96)	0.57 (2.63)	0.003*
p16+ Granulocytes %	2.49 (4.57)	1.37 (4.24)	3.16 (5.35)	3.57 (10.74)	0.178
p53+ Lymphocytes, %	0.04 (0.04)	0.04 (0.08)	0.04 (0.04)	0.05 (0.02)	0.952
p53+ Monocytes, %	0.08 (0.07)	0.07 (0.08)	0.1 (0.09)	0.07 (0.06)	0.382
p53+ Granulocytes %	5.47 (8.3)	4.01 (4.69)	7.05 (8.88)	8.35 (9.56)	0.052
p21+ Lymphocytes, %	1.8 (5.83)	3.31 (5.38)	1.79 (6.95)	0.91 (5.38)	0.314
p21+ Monocytes, %	1.53 (3.75)	1.11 (3.47)	1.4 (2.17)	3.37 (4.28)	0.771
p21+ Granulocytes %	4.41 (9.28)	0.78 (1.67)	5.75 (7.58)	11.17 (27.55)	< 0.001*
Cytotoxic T cells, %	33.92 (22.49)	57.67 (28.25)	29.53 (10.93)	33.81 (14.82)	< 0.001*
T helpers, %	41.67 (15.96)	28.49 (20.22)	45.88 (9.82)	42.98 (14.0)	0.016*
Th1, %	16.19 (6.61)	15.97 (7.26)	16.34 (6.51)	15.95 (10.93)	0.998
Th2, %	7.13 (8.83)	9.39 (13.32)	5.81 (3.34)	6.47 (7.02)	0.153
Th17, %	8.31 (7.29)	3.91 (2.2)	8.83 (5.08)	9.68 (10.93)	0.015*
T reg, %	7.52 (1.74)	7.63 (2.71)	7.39 (1.77)	7.51 (2.02)	0.278
TFH, %	11.31 (6.26)	15.04 (8.04)	9.8 (4.25)	14.25 (6.38)	0.003*

Values are presented as median (IQR). Percentages are expressed relative to the corresponding parent population: p16^+^, p21^+^, and p53^+^ subsets are shown as % of lymphocytes, monocytes, and granulocytes, respectively; cytotoxic and helper T cells are expressed as % of total T cells; Th1, Th2, Th9, Th17, Treg, and TFH subsets are expressed as % of CD4^+^ T cells.

**Figure 3:**
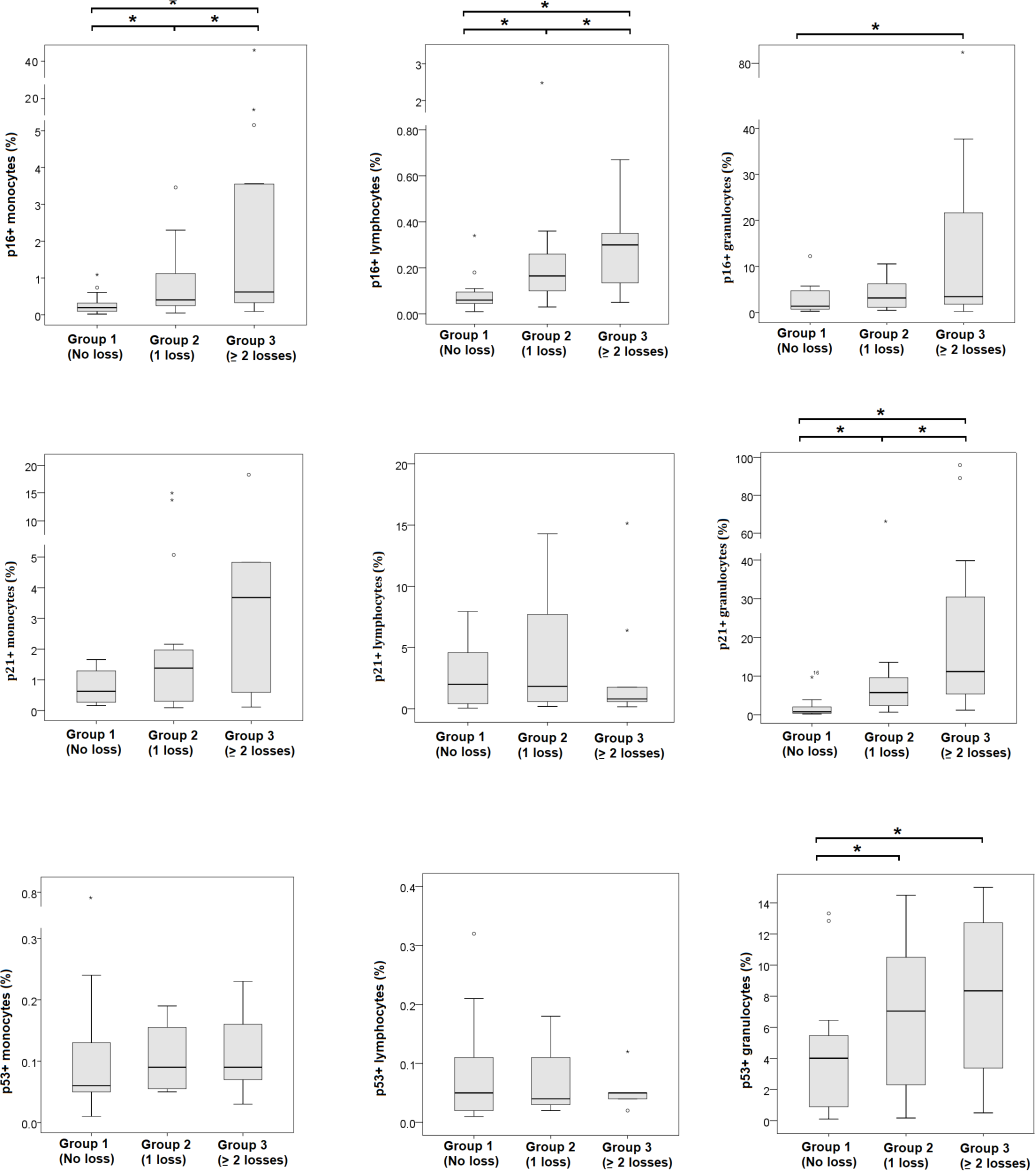
Distribution of senescent immune cell frequencies in peripheral blood during early pregnancy according to reproductive history. Percentages of p16^+^, p21^+^, and p53^+^ lymphocytes, monocytes, and granulocytes are shown as box plots and compared across groups with no prior miscarriage (Group 1), one miscarriage (Group 2), or recurrent pregnancy loss (Group 3). All data points are displayed. Where required, a segmented (broken) y-axis was used to preserve visibility of both the interquartile range and extreme values. Statistical comparisons were performed using the Kruskal–Wallis test, with significant differences indicated in the figure. Asterisks (*) denote statistically significant differences between the indicated groups (p < 0.05), as shown by the brackets in the figures.

**Figure 4:**
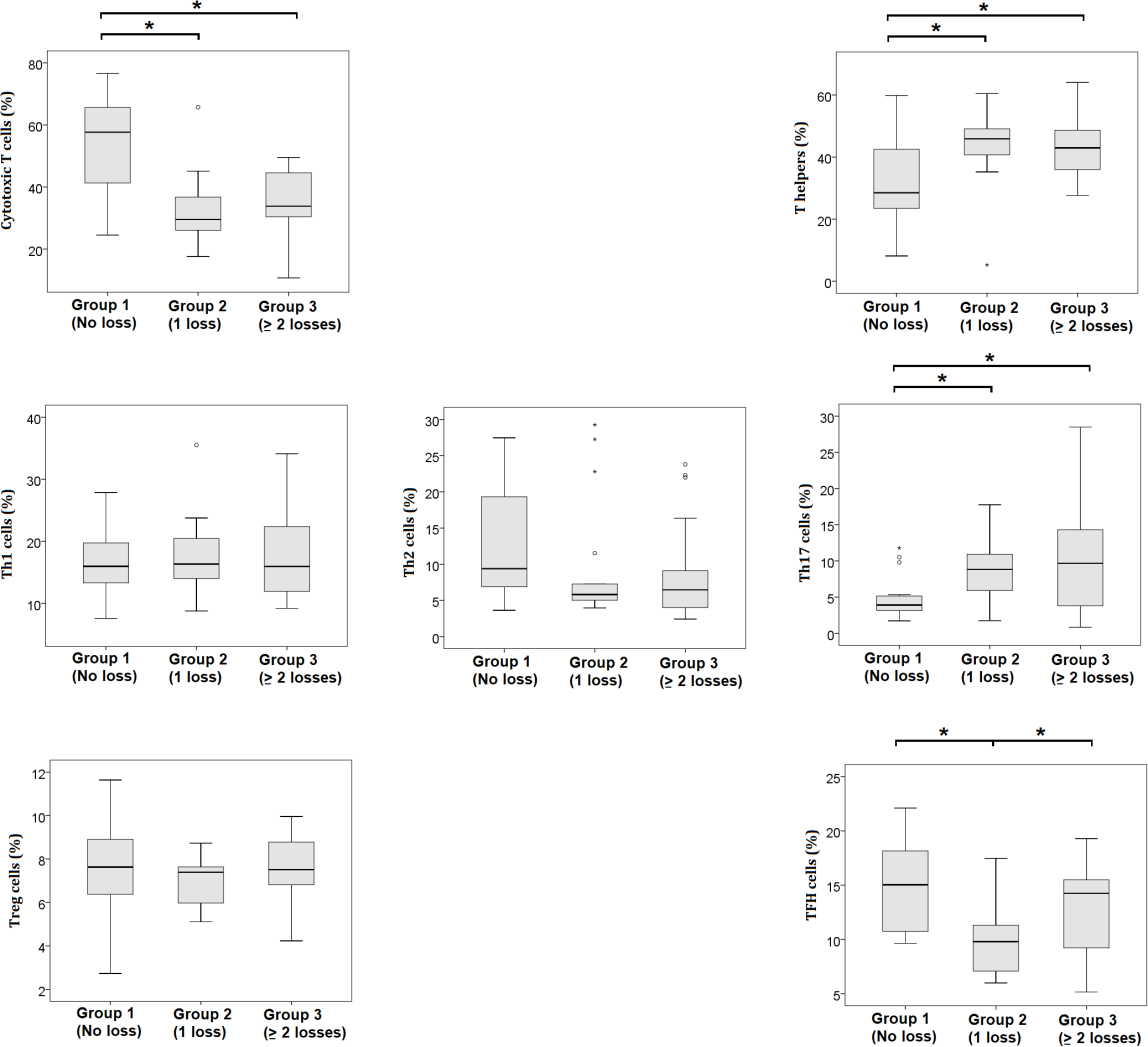
Distribution of peripheral T-cell subsets during early pregnancy, shown as box plots by reproductive history. Percentages of cytotoxic T cells, T helper cells, Th1, Th2, Th17, Treg, and TFH subsets are compared across groups with no prior miscarriage (Group 1), one miscarriage (Group 2), or recurrent pregnancy loss (Group 3). Asterisks (*) denote statistically significant differences between the indicated groups (p < 0.05), as shown by the brackets in the figures.

A gradual increase in the percentage of senescent immune cells was observed in association with the number of previous pregnancy losses (**Figure 3**). The median percentage of p16+ monocytes was 0.20% in controls, 0.41% in women with one prior loss, and 0.57% in the RPL group (p = 0.03). Similarly, p16+ lymphocytes increased from 0.06% (controls) to 0.17% (one loss) and 0.35% (RPL) (p < 0.01). For granulocytes, p21+ expression rose from 0.78% in the control group to 5.75% and 11.17% in the one-loss and RPL groups, respectively (p = 0.04). A similar trend was observed for p53+ granulocytes, increasing from 4.01% to 7.05% and 8.35% (p = 0.05). In addition to senescence markers, significant differences were also found for CD8^+^ cytotoxic T cells (median: 57.7%, 33.9%, 33.8%; p < 0.001), CD4^+^ helper T cells (28.5%, 41.7%, 43.0%; p = 0.016), Th17 cells (3.9%, 8.8%, 9.7%; p = 0.015), and TFH cells (15.0%, 9.8%, 14.3%; p = 0.003) across the three groups (**Table 2**). These group-level differences are further illustrated in **Figure 4**, which depicts the distribution of individual values for each T-cell subset.

**Figure 5:**
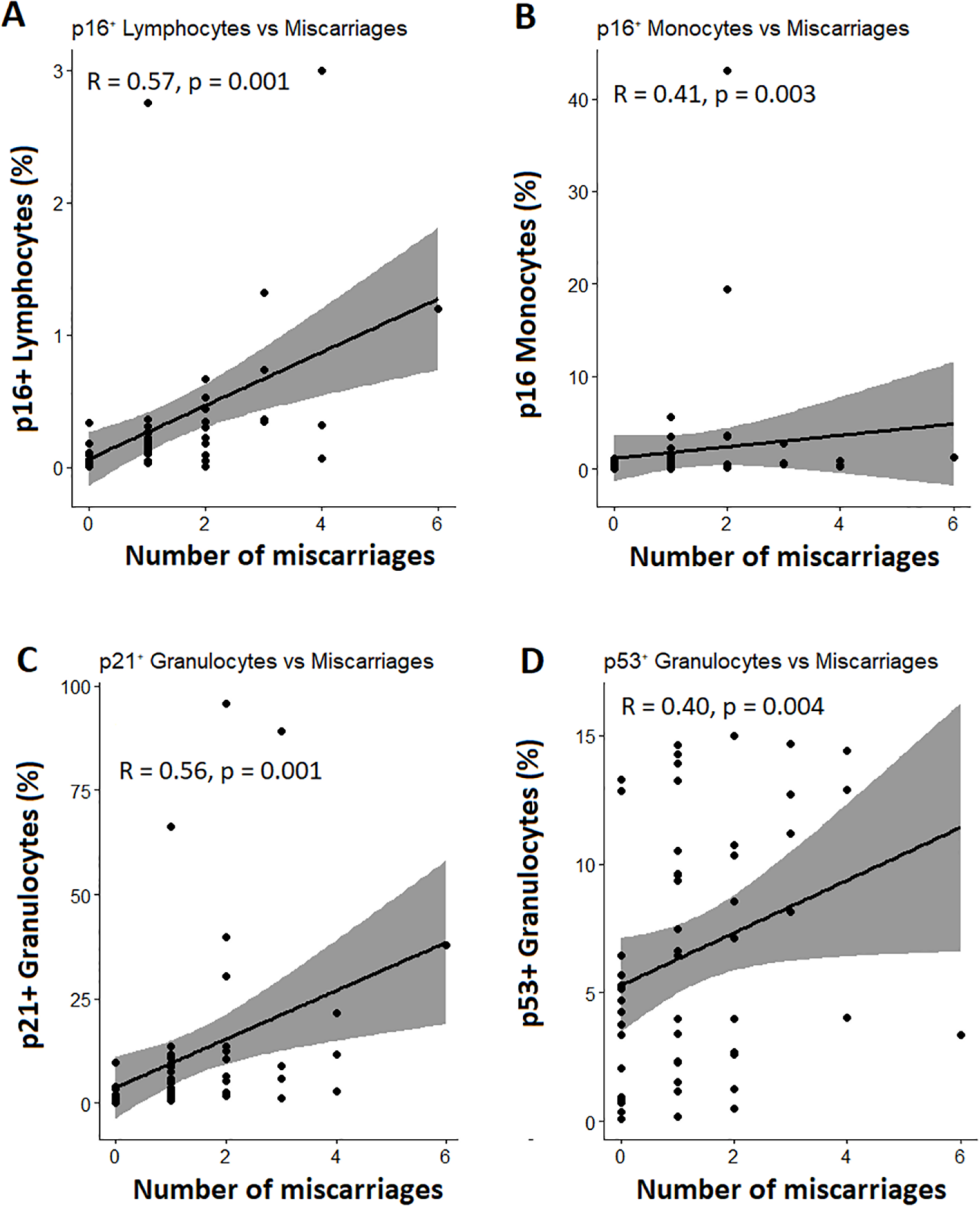
Significant associations between senescent immune cell subsets and the number of previous miscarriages. Panels A–D show scatter plots illustrating significant correlations between miscarriage number and **(A)** p16^+^ lymphocytes, **(B)** p16^+^ monocytes, **(C)** p21^+^ granulocytes, and **(D)** p53^+^ granulocytes. Spearman correlation coefficients (R) and corresponding p-values are indicated in each panel. Regression lines with 95% confidence intervals are shown.

The senescence-related group differences were further supported by a Spearman analysis (**Figure 5**), which showed significant positive associations between the number of previous miscarriages and the percentages of senescent immune cells. The strongest correlations were observed for p16^+^ lymphocytes (R = 0.57) and p16^+^ monocytes (R = 0.41), followed by p21^+^ (R = 0.56) and p53^+^ (R = 0.40) granulocytes (p < 0.05 for all).

**Figure 6:**
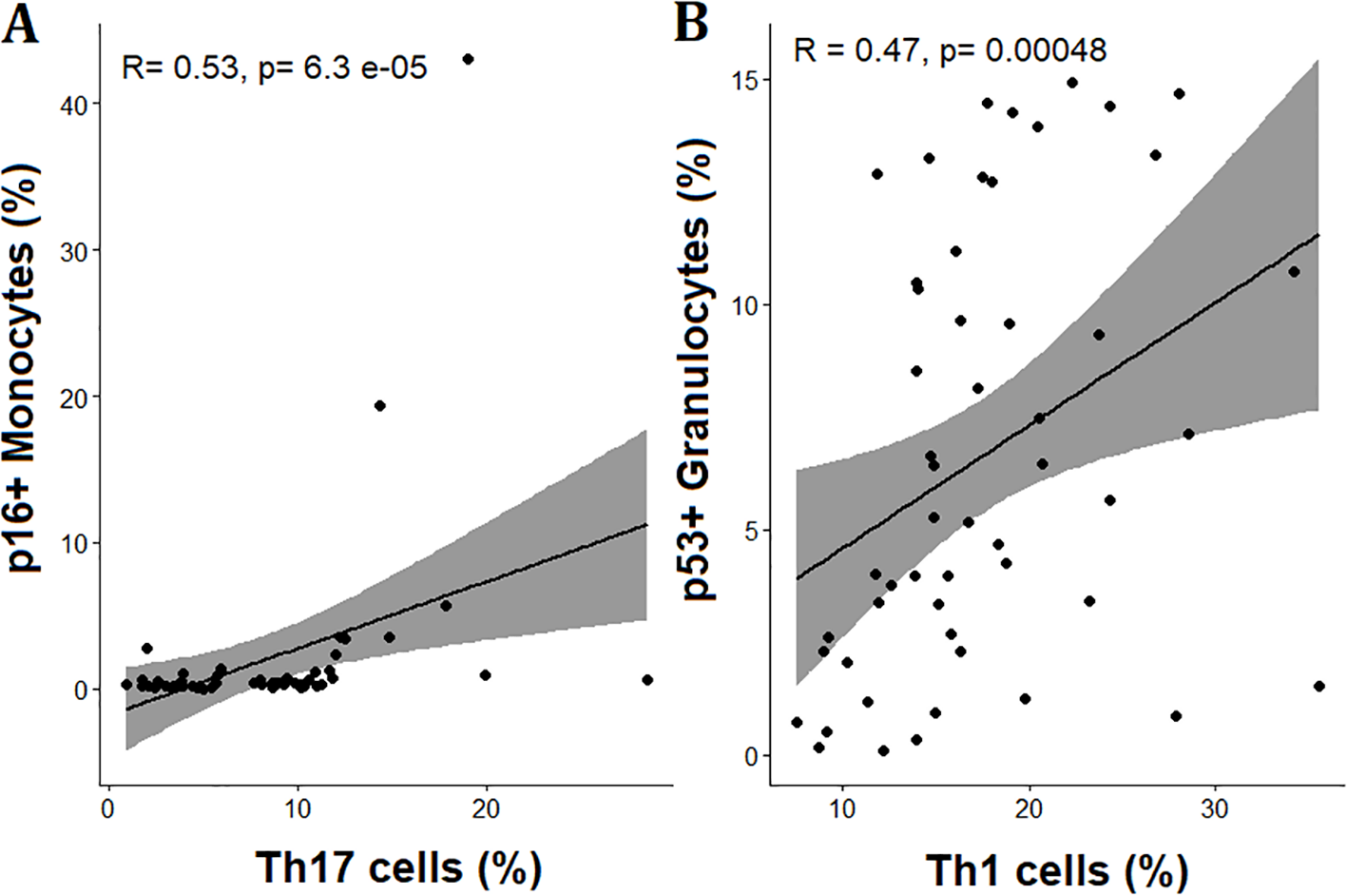
Significant associations between senescent immune cell subsets and proinflammatory T-cell populations in early pregnancy. Panels A and B show scatter plots illustrating significant correlations between **(A)** p16^+^ monocytes and Th17 cells, and **(B)** p53^+^ granulocytes and Th1 cells. Spearman correlation coefficients (R) and corresponding p-values are indicated in each panel. Regression lines with 95% confidence intervals are shown.

A separate exploratory correlation analysis across the full cohort revealed specific associations between senescent immune cell subsets and certain proinflammatory lymphocyte populations (**Figure 6**). The percentage of peripheral p16+ monocytes correlated positively with the abundance of Th17 cells (R = 0.53, p < 0.01) and p53+ granulocytes with the abundance of Th1 cells (R = 0.47, p < 0.01). No consistent correlations were observed between other senescence markers and the remaining T cell subsets.

## Discussion

Our findings highlight the involvement of peripheral senescent immune cells in early pregnancy and their links to proinflammatory T-cell subsets in women with recurrent pregnancy loss (RPL). In this study, we observed significant increases in the percentages of p16^+^ monocytes and lymphocytes, as well as p21^+^ and p53^+^ granulocytes in the pregnancy loss groups. Moreover, this change correlated with the number of previous miscarriages. This may reflect cumulative senescence-associated immune dysfunction that contributes to adverse reproductive outcomes. In addition to these quantitative increases, we also observed distinct qualitative differences in the organization of senescence markers expression across leukocyte subtypes. Taken together, the observed dose–response pattern and the altered marker organization support the hypothesis that repeated pregnancy loss is not only associated with quantitative immune senescence, but also may actively drive qualitative reprogramming of leukocyte cell types toward a senescence-associated, proinflammatory phenotype. These findings imply that immune aging may not be a static condition but rather a dynamic process influenced by obstetric history, potentially predisposing to further reproductive complications.

In the control group without previous pregnancy loss, a consistent pattern of p21 > p16 > p53 was present across monocytes, lymphocytes, and granulocytes, suggesting a tightly regulated, physiologic senescence hierarchy in early pregnancy. It is well known that p21 is a key mediator of p53-dependent cell cycle arrest, promoting a more controlled and less inflammatory form of senescence, characterized by a distinct secretome and SASP profile compared to the p16 pathway (20). In contrast, senescence driven by p16 is associated with irreversible arrest, sustained SASP secretion, and stronger proinflammatory signaling. Murine models have demonstrated distinct immune consequences following clearance of p21+ vs. p16+ senescent cells, further supporting the functional divergence between these two major pathways (21).

This physiological organization was progressively disrupted in women with prior pregnancy losses, where p16 and p53 expression became more prominent and less ordered across immune subsets. This shift may reflect a transition toward stress-induced senescence, particularly within the myeloid compartment. Granulocytes, in particular, showed the highest p16 expression in affected women. Such accumulation of senescent myeloid cells may contribute to a dysregulated systemic immune environment, potentially priming endometrial or decidual tissues for impaired trophoblast–maternal interaction. Recent work shows that neutrophils can acquire senescent features (including p21 and p16 expression) *in vivo* after injury and in long-term inflammation, supporting a myeloid bias toward stress-induced senescence (22, 23). Such a shift is consistent with previous findings demonstrating that myeloid cells can acquire p16-associated senescence under inflammatory conditions, contributing to innate immune dysfunction (24, 25). Mechanistically, p16^+^ senescent cells can upregulate PD-L1, facilitating immune evasion and persistence in inflammatory tissues. This effect may stabilize senescent myeloid pools in susceptible pregnancies (26). These maladaptive responses could impair maternal–fetal immune tolerance and promote pregnancy complications.

Moreover, the exploratory Spearman analyses revealed significant positive correlations between p16+ monocytes and Th17 cells, as well as between p53+ granulocytes and Th1 cells. Both associations were observed across the full cohort, suggesting a potential link between senescence-related myeloid dysfunction and proinflammatory adaptive immune responses. These axes are in agreement with contemporary RPL immunology, where Th17/Treg imbalance and Th1-skewing are repeatedly implicated in implantation failure and miscarriage risk (27). The lineage specificity observed here aligns with reports that p16, p21 and p53 vary across T cells, B cells, monocytes and granulocytes, as well as across differentiation stages, supporting subset-level rather than bulk blood analyses (11). In addition to senescence-related changes, we also observed significant group-level differences in broader T-cell populations, including cytotoxic cells, helper cells, Th17, and TFH subsets. These shifts are consistent with earlier studies reporting altered CD4/CD8 ratios and enhanced cytotoxic T-cell frequencies in women with RPL (28), as well as disturbed Th17/Treg balance (29) and increased TFH populations contributing to abnormal B-cell help and autoantibody responses in miscarriage (30). While it was not the main focus of our analysis, these findings showed that pregnancy loss is accompanied by multifaceted immune remodeling extending beyond cellular senescence.

Our study did not resolve senescence within specific T cell subsets (e.g., CD4^+^ versus CD8^+^), which are known to differ in their susceptibility to senescence (31). This represents a limitation that future studies should address by integrating subset-level analyses to refine the understanding of lymphocyte senescence in the context of pregnancy loss. Methodologically, our subset-resolved, intracellular flow-cytometry approach is consistent with prior blood-based work on cell-cycle regulators; reliable detection of nuclear antigens such as p53 requires optimized fixation/permeabilization and antibody clone selection, as recommended for human blood assays.

Although p16, p21, and p53 are widely used and well-validated markers of senescence-associated cell cycle arrest, they represent only one dimension of the broader senescence phenotype. Additional hallmarks, including senescence-associated β-galactosidase activity, mitochondrial dysfunction, metabolic reprogramming, and the senescence-associated secretory phenotype (SASP), characterized by increased production of proinflammatory mediators such as IL-6, may provide complementary functional insight into senescent immune cell states. However, many of these features are not readily amenable to standardized, subset-resolved, high-throughput flow-cytometric assessment in clinical peripheral blood samples. Our approach therefore prioritizes robust detection of core senescence regulators that enable comparative analysis across immune subsets in human pregnancy. Future studies integrating functional assays or single-cell approaches will be important to further refine the characterization of senescent immune states in the context of pregnancy loss.

As with most clinical studies, subclinical immune activation in the absence of overt infection cannot be entirely excluded, although no participant showed clinical evidence of active infection at the time of sampling. Another limitation of the present study is the relatively small sample size within each subgroup, which may limit statistical power for detecting more subtle effects and restrict the generalizability of the findings. Furthermore, other potential confounding factors, such as comorbid conditions or prior immune-modulating exposures, were not systematically assessed and therefore cannot be entirely excluded.

An additional limitation of this study is that pregnancy outcome after inclusion was not analyzed. Blood samples were collected at an early gestational stage, prior to or concomitant with individualized supportive clinical interventions implemented as part of routine care for women with a history of pregnancy loss. Consequently, subsequent pregnancy outcome would be influenced by post-sampling clinical interventions and would not accurately reflect the baseline immune senescence state assessed here. The present work therefore focuses on immune senescence profiles at a defined early pregnancy time point rather than on prediction of ongoing pregnancy success. Future prospective studies with standardized management protocols and predefined outcome assessment will be required to address the prognostic relevance of peripheral immune senescence.

An additional contextual consideration is that all immune measurements were performed during an ongoing early pregnancy, at a stage characterized by successful implantation and detection of fetal cardiac activity. Thus, the observed senescence-associated immune alterations reflect immune states present during early gestation rather than preconceptional baseline profiles. It remains an open question whether similar senescence patterns are already established in non-pregnant women with a history of pregnancy loss, or whether they emerge or become amplified in response to early gestational immune adaptation. Future studies directly comparing non-pregnant women with prior pregnancy loss to early pregnant cohorts will be essential to disentangle pre-existing immune senescence from pregnancy-induced immune remodeling.

Our observations are consistent with accumulating evidence that Th17 cells contribute to the pathogenesis of recurrent miscarriage and implantation failure. Increased circulating Th17 cells have been reported in women with unexplained recurrent pregnancy loss and are thought to contribute to a proinflammatory environment detrimental to fetal tolerance (32). Senescent monocytes may contribute to Th17 expansion via secretion of proinflammatory cytokines, such as IL-6 and IL-1β, which are known to support Th17 differentiation (33, 34). Our data support this hypothesis, showing a positive Spearman correlation between p16^+^ monocytes and Th17 cells in the full cohort.

**Figure 7:**
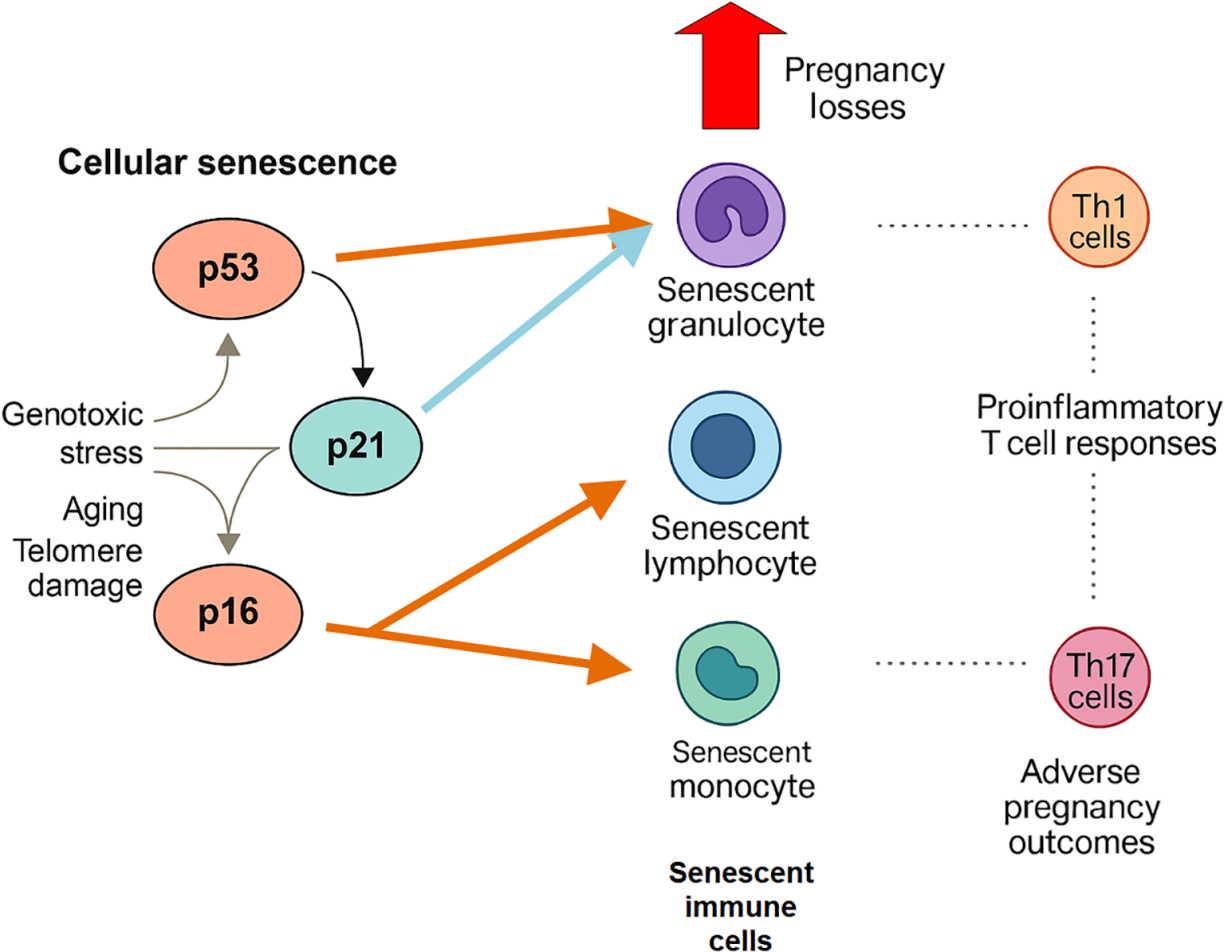
Putative model linking peripheral immune senescence to maladaptive inflammatory responses in early pregnancy. The schematic summarizes a hypothetical interaction framework derived from cross-sectional correlation data and published literature. The model is intended to provide conceptual context and to generate testable hypotheses for future mechanistic studies, rather than to imply direct causality.

The observed correlation between p53+ granulocytes and Th1 cells may reflect a broader senescence-driven skewing of adaptive immunity toward Th1 dominance, a phenotype previously associated with immune-mediated pregnancy loss (35). Decidual mechanisms normally limit Th1/Tc chemotaxis; disruption of this moderation has been documented in human decidua and may amplify Th1-driven risk (36). Interestingly, IFN-γ and TNF-α, key Th1 cytokines, have been shown to induce cellular senescence *in vitro* (37), raising the possibility of a bidirectional feedback loop between inflammatory T cells and senescent innate immune cells. These observations, together with our correlation data, are integrated in **Figure 7** as a working model of senescence–inflammation crosstalk in recurrent pregnancy loss. This model is deliberately speculative and is meant to generate hypotheses for future mechanistic studies rather than to imply direct causality.

While the primary aim of this study was to characterize innate immune senescence in relation to pregnancy loss, we additionally explored associations with selected T helper cell subsets based on a mechanistic rationale. Senescence-associated secretory phenotypes in monocytes may promote Th17 polarization, while granulocyte-driven inflammation has been linked to Th1-skewed immune responses in reproductive disorders. In line with this framework, we observed associations between p16^+^ monocytes and Th17 cells, and between p53^+^ granulocytes and Th1 cells. However, these correlations may be influenced by a limited number of individuals with higher values and should therefore be interpreted cautiously. Larger studies using outlier-robust approaches will be required to validate these observations.

## Conclusion

This study provides novel evidence that peripheral immune senescence in early pregnancy increases progressively with the number of previous miscarriages, involving distinct molecular pathways. The most consistent change was the accumulation of p16^+^ monocytes and lymphocytes, accompanied by significant increases in p21^+^ and p53^+^ granulocytes. These patterns suggest that both p16-associated and alternative senescence programs may contribute to reproductive failure. Positive correlations between p16^+^ monocytes and Th17 cells, and between p53^+^ granulocytes and Th1 cells, indicate that senescent myeloid populations may promote proinflammatory adaptive responses. Taken together, these findings highlight senescent immune cells as candidate biomarkers and potential contributors to immune maladaptation in women at risk of pregnancy loss. Future studies should clarify the underlying mechanisms and explore whether interventions aimed at modulating senescence could improve maternal immune adaptation and support successful pregnancy.

## Data Availability

The datasets presented in this study can be found in online repositories. The names of the repository/repositories and accession number(s) can be found below: https://zenodo.org/records/17090816?token=eyJhbGciOiJIUzUxMiJ9.eyJpZCI6IjI5ZmVmNzgxLTAwMDAtNGJmMi1iYTdmLWFkMWQwNjQ4NTlkYiIsImRhdGEiOnt9LCJyYW5kb20iOiJkYmExYzAwODE3NmI0ZWY4YTI0MzQ1Zjc3MjJmZTFiZiJ9.XSe-RdT1VY00B6Wix2JQpOpfPZ7ZkngMOOWh5_UBLERkOcyw_f9mbY6XJS2U30VPy72X5nqoviO0xNCqczsxAg.
